# Dyadic movement synchronization while performing incongruent trajectories requires mutual adaptation

**DOI:** 10.3389/fnhum.2014.00461

**Published:** 2014-06-24

**Authors:** Tamara Lorenz, Björn N. S. Vlaskamp, Anna-Maria Kasparbauer, Alexander Mörtl, Sandra Hirche

**Affiliations:** ^1^Experimental Psychology, Ludwig-Maximilians UniversityMunich, Germany; ^2^Institute for Information-Oriented Control, Technische Universität MünchenMunich, Germany; ^3^Philips ResearchEindhoven, Netherlands; ^4^Institute of Psychology, Rheinische Friedrich-Wilhelms UniversitätBonn, Germany

**Keywords:** joint action, movement synchronization, obstacle avoidance, movement interference, movement coordination, action timing

## Abstract

Unintentional movement synchronization is often emerging between interacting humans. In the present study, we investigate the extent to which the incongruence of movement trajectories has an influence on unintentional dyadic movement synchronization. During a target-directed tapping task, a participant repetitively moved between two targets in front of another participant who performed the same task in parallel but independently. When the movement path of one participant was changed by placing an obstacle between the targets, the degree of their unintentional movement synchronization was measured. Movement synchronization was observed despite of their substantially different movement trajectories. A deeper investigation of the participant's unintentional behavior shows, that although the actor who cleared the obstacle puts unintentional effort in establishing synchrony by increasing movement velocity—the other actor also unintentionally adjusted his/her behavior by increasing dwell times. Results are discussed in the light of joint action, movement interference and obstacle avoidance behavior.

## Introduction

Synchronization is a phenomenon which naturally emerges across species and systems (Strogatz, 2003). Between humans, almost everybody has experienced that when walking next to another person, one automatically synchronizes walking pace (van Ulzen et al., 2008; Nessler and Gilliland, 2009). Interestingly, people synchronize their movements as soon as they exchange sensory information (Issartel et al., 2007) and it seems that if visual information on the other's movements is available, synchronization is inevitable (Schmidt and O'Brien, 1997). Previously, synchronization was studied in numerous tasks like for example tapping (Schöner et al., 1986; Konvalinka et al., 2010), pendulum swinging (Richardson et al., 2008), walking (Nessler and Gilliland, 2009), rocking in chairs (Richardson et al., 2007) or drumming (Kirschner and Tomasello, 2009). These studies showed that people tend to adapt to each other and synchronize their movements to either an in-phase relation (being at the same stage of the movement at the same time) or an anti-phase relation (being at the opposite stage of the movement at the same time).

Yet little is known about the requirements for movement synchronization to occur. Interaction partners need to be able to make movements producing equal rhythm, which requires similar temporal and spatial abilities. However, there may be more subtle complications. If humans act alone, their movements are believed to follow certain efficiency criteria such as minimization of movement time or required energy (Engelbrecht, 2001) [although principles of motor control are not completely understood yet (Latash et al., 2010)]. Accordingly, one would expect these efficiency criteria to be a necessary requirement during interaction, and thus also for movement synchronization. Therefore it is remarkable that interpersonal synchronization is even established if the individual's minimization criterion is violated. One example is provided by Richardson et al. (2007) who had participants rocking in chairs at their own preferred rate. When their chairs were manipulated to have differing natural frequencies, the coherence—as a measure of entrainment or coupling strength—was decreased compared to the case when both participants were rocking in chairs with the same natural frequencies. This potentially resulted from the higher effort to maintain a phase-locked frequency relation if the natural frequencies of the systems differ. But although the required energy was higher for one person, synchronization still emerged unintentionally.

However, Richardson et al. only manipulated the natural frequencies of the chairs, while the temporal and spatial constraints were still equal for both participants: people were rocking in identical chairs. Thus, the trajectories performed while rocking were still the same. Here, a particularly interesting—and so far untested—situation arises: does unintentional synchronization still emerge when movement trajectories are different?

If synchronization emerges in such a situation, this requires an unintentional adjustment of movements from at least one person. With this however, additional complications arise: it is known that when people watch movements different to those performed by themselves (incongruent behavior), a so called *interference effect* can be observed (Brass et al., 2001; Kilner et al., 2003; Sebanz et al., 2003; Stanley et al., 2007). The interference effect increases movement onset and reaction times in response to spatial incompatibility (Brass et al., 2001; Sebanz et al., 2003), but it also affects movement trajectories (Kilner et al., 2003; Stanley et al., 2007). It is suggested that while performing the own action, a simultaneous activation of the human equivalent to the mirror neuron system (Rizzolatti et al., 1996; Rizzolatti, 2005) causes some kind of *motor contagion* (Blakemore and Frith, 2005). By observing somebody else's action and activating one's representation of it, the own action is facilitated. However if the observed action does not match the representation of the own executed movements, this might create additional load as incorrect motor programs have to be inhibited. For the actual execution of movement this means that people tend to unintentionally mirror the movements they see, while they actually intentionally try to perform a different movement. This conflict is hypothesized to cause the deviation. Nevertheless, visual information exchange was also found to be crucial for unintentional synchronization to occur (see Richardson et al., 2005). Thus, in a situation in which trajectories are incongruent and may interfere due to visual information exchange, an interesting question is whether movement synchronization would still emerge.

In order to answer this question we introduce a study in which participants performed repetitive target-directed arm movements. From previous work it is known that in these tasks, movement synchronization occurs rapidly when participants can make congruent movements (Lorenz et al., 2011; Mörtl et al., 2012). Thus, for the study at hand we exploit a similar experimental setup. An obstacle is put in one participant's way—which causes an adjustment of movement trajectories for the actor who has to move around it.

Obstacle avoidance as a single action is characterized by deviations which cause increased movement times or a decrease in movement speed respectively (Tipper et al., 1997; Coppard et al., 2001; Castiello, 2003; Chapman and Goodale, 2008; Menger et al., 2012). During joint action this can also have implications for synchronization. If one actor has to move around an obstacle, movements become incongruent in space and might therefore also be incongruent in time. Therefore, in this study we ask whether synchronization still emerges if movement trajectories of two individuals are incongruent in space and therefore also do not match in time—and which strategies are applied to establish it.

## Materials and methods

In order to access participants' movement synchronization behavior during incongruent conditions, a dyadic target-directed tapping-task was developed in which one participant had to clear an obstacle, which enforces the performance of a different trajectory. The experiment was approved by the ethics committee of the faculty for psychology and pedagogics of the LMU and conducted in accordance with the Declaration of Helsinki.

### Participants

Ten dyads (20 people, 7 female) took part in the experiment. Their age ranged from 21 to 45 years (*M* = 26.6 years). Participants were recruited from a local (LMU Munich) participant database and paid 8 Euro/hour for participation. All participants were right handed and had normal or corrected to normal vision. Prior to the experiment, all participants were provided with written instructions and signed written consent.

### Experimental setup

**Figure 1:**
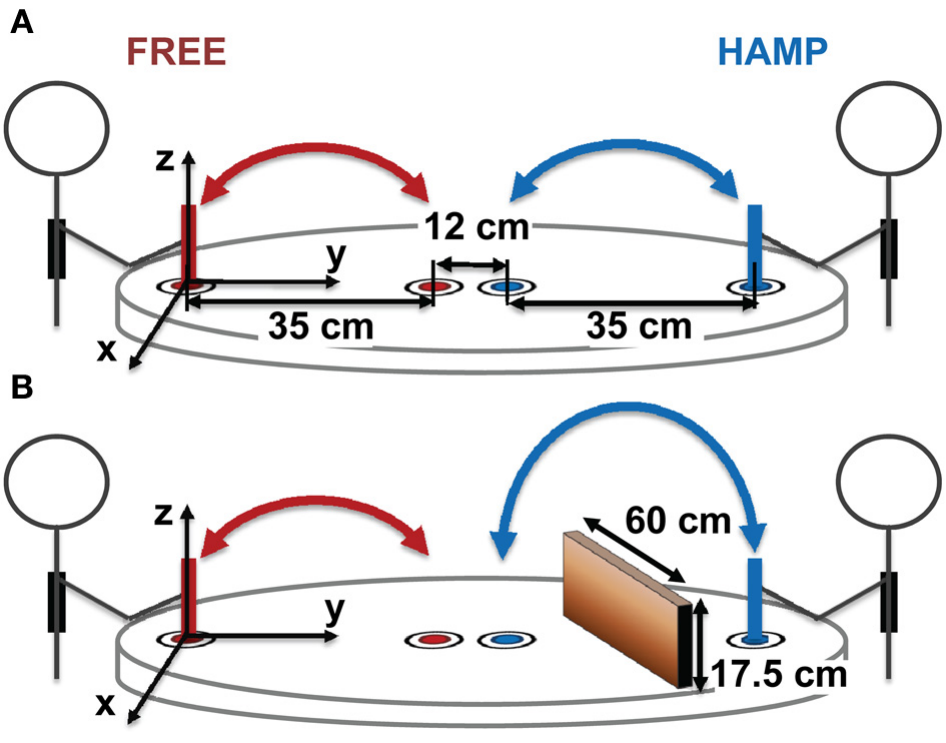
**Experimental Setup. (A)** configuration without obstacles, **(B)** configuration with obstacle for HAMP. Both participants repetitively moved a stylus with their right hand from one target position (close to them) to another (in the middle of the table) and back.

Each dyad sat at a round table (radius = 0.575 m) facing each other, see Figure 1. Four colored target cycles with a diameter of 8 mm were marked on the table. Each target was surrounded by a white area (diameter 60 mm) to increase the saliency of the colored target. Every actor was assigned with two targets of the same color on his or her side of the table. Targets of the same color were 0.35 m apart while the distance of the different colored targets in the center of the table was 0.12 m. The target which was located closer to the body was defined to be the start. Both participants were equipped with a stylus (height 13.5 cm) which had the same diameter as the target and instructed to power-grasp it with their right hand. Additionally they wore SONY stereo headphones (MDR-XD200) with a connection to the control PC. Headphones were used to trigger the beginning and end of a trial via a short acoustical beep. Movements were recorded using an infrared tracking system (PTI Visualeyez II VZ4000). Wired LEDs were attached to the top of each stylus and tracked with a camera bar mounted at the ceiling. The tracking system had an online sampling rate of 30 Hz for calculating the start signal delays (see section Procedure) and an offline sampling rate of 200 Hz for data recording.

During obstacle present trials, a vertical obstacle (height: 0.175 m, width: 0.6 m) was positioned in the workspace of one participant. It was placed orthogonally to movement direction at half the distance between targets, see Figure 1.

Note that only one participant of the dyad was *hampered* by an obstacle in 50% of all trials (HAMP). The other participant always acted without an obstacle in his/her *free* movement path (FREE). Note also that both participants were always able to see all four targets, even when the obstacle was present.

### Procedure

Before the beginning of each trial, participants rested in the closer target with their stylus oriented orthogonally to the table. They were instructed to begin moving with the acoustical start signal, lift the stylus from the individual start position to the further apart target, tap on the target, move back and tap on the start position again. This procedure was repeated continuously. After both participants performed at least 10 cycles each, the tracking system automatically triggered an acoustical stop signal. Note that participants were not informed about the cycle counter. Also, only start and stop signals were provided. There was no synchronization signal or any other rhythmical guideline. Moving forward, tapping, moving backward and tapping again will be considered as one *cycle*.

Instruction remained the same when an obstacle was present. However, participants were told not to touch or collide with the obstacle. Thus, the obstacle required lifting the stylus over it and with this a change of trajectory was induced.

In order to avoid synchronization to emerge only because of a simultaneous start trigger, different cycle-dependent timings of start signals were calculated online which resulted in three different *start delays*: (1) *zero-cycle*: the start signal was presented simultaneously for both participants, (2) *quarter-cycle*: the start signal for the follower was presented when the beginner had passed half the way to the second target; (3) *half-cycle*: the start signal for the follower was presented when the beginner had reached the second target.

Each dyad performed 12 sets of 6 trials, which results in 72 trials (720 cycles) in total. Within each set, start delay was kept the same while the *configuration* (*congruent*: both participants did not have to clear the obstacle or *incongruent*: one participant had to clear the obstacle) was randomly assigned for each trial and randomized within each set. In sets with start delays quarter- and half-cycle, being beginner was also randomly assigned to one participant and counterbalanced within each set.

Note that participants were naïve as to the purpose of the experiment. Participants were not instructed in any way to synchronize their movements or to adapt their movements to each other. This allowed us to explore if and how synchronous behavior emerges naturally.

### Data preparation

Movement data was recorded in 3-dimensions over time, i.e., for every data point there is information on when it was recorded and the position in x, y and z with origin at the closer target, see Figure 1. Here, x refers to horizontal deviation of the movements, y to the progress of forward and backwards movements and z to the elevation above the table surface. Data was prepared, processed and segmented in Matlab; statistical analyses were performed in SPSS.

For every trial, movement onset was calculated as the first time the z-component of the movement crossed 0.005 m. Data analysis only considered movement data after the latest movement onset per trial. Thus, data analysis was always on joint action, not on single action.

**Figure 2:**
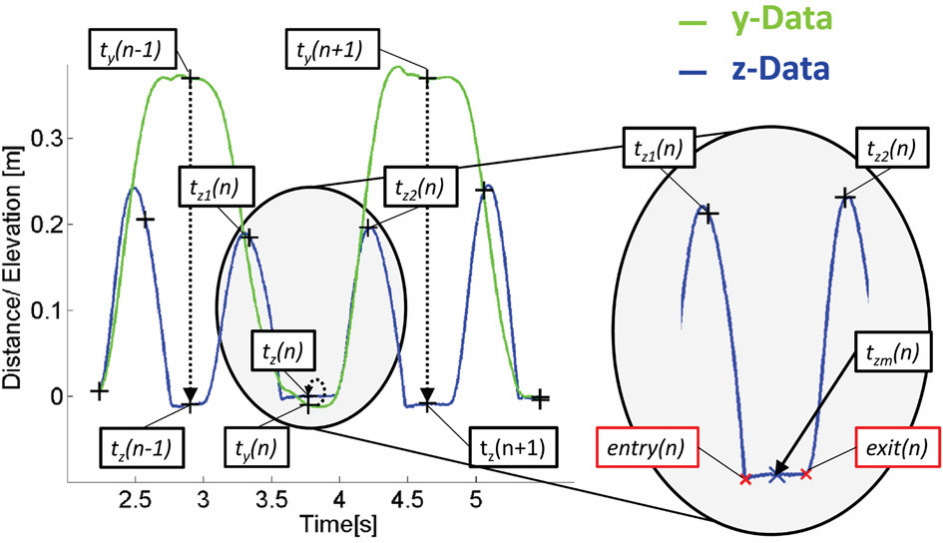
**Principle of data segmentation shown on exemplary movement data of the free actor in the congruent configuration**. The y-component (green) is used for tapping event detection while dwell time and movement time are determined by the isochronic z-component (blue), see text for more detailed explanation.

For being able to look at the emerging behavior and potential adaptation process, in every trial movement data of each person was segmented into half-cycles (forwards and backwards segments). For segmentation, the times *t*_*y*_(*n*) of the trajectory's y-component inflection points were determined, where *n* ϵ *N* counts the amount of tapping actions per trial, see Figure 2. As the y-component describes the movement direction between target points, each inflection point represents a turn in direction and thus indentifies a tapping time. From the timestamp of *t_y_*(*n*) = *t_z_*(*n*) a temporal window defined by *t*_*z*2_(*n*) and was *t*_*z*2_(*n*) determined as

(1) $$t_{z1}(n)=\left(t_{z}(n-1)+t_{z}(n)\right)/2$$

(2) $$t_{z2}(n)=\left(t_{z}(n)+t_{z}(n+1)\right)/2$$

Following this, *z*_*m*_(*n*) was determined as the trajectories z-component value at time *t*_*zm*_(*n*) which was calculated as

(3) $$t_{zm}(n)=\left(t_{z1}(n)+t_{z2}(n)\right)/2$$

The actual entry and exit to the dwelling phase of the *n*th tapping was then determined by detecting the closest intersection of the z-component of the trajectory with *t*_*zm*_(*n*) + 0.005m before and after *t*_*zm*_(*n*). Data was visually checked and all trials not captured with the automated procedure were manually corrected. Trials in which obstacles were hit and/or an early/late start was detected, were excluded. Overall this resulted in 88% valid trials.

### Data analysis and dependent variables

Data Analysis was performed in three steps. First, it was determined if the spatial manipulation of the experiment was successful, i.e., if HAMP extended his/her trajectory during obstacle present trials as measured by the *path length* (PL) in 3-dimensional space. During this step it was also checked whether the obstacle for HAMP had an impact on the PL of the free actor.

In a second step, the emergence of synchronization was checked over all possible conditions. Therefore, phase data was calculated as described in Mörtl et al. (2012) using the Hilbert-transform (spectral method). From the resulting phase data, the dyadic phase difference (*relative phase*) was calculated for every data point per trial. This resulting relative phase data was clustered into nine pi/9 (20°) relative phase regions (Schmidt and O'Brien, 1997; Richardson et al., 2007; Coey et al., 2011) by counting and accumulating the times the relative phase was in one of the defined regions. Of the accumulated data, percentages were calculated for each condition, see **Figure 4**. For creating a reference, data from non-interacting participants was combined and the resulting phase relation was determined. Therefore each participant's data was combined with all data from all other participants within the respective conditions and clustered as described previously. Here, participants never interacted with each other. Therefore this data should reflect a case in which no synchronization emerges.

Furthermore, for having some information on the quality of the synchronization, the *cross spectral coherence* was calculated as

(4) Coherence=1−CV

where CV is the circular variance of the relative phase over time (Kreuz et al., 2007). The coherence can vary between 0 and 1. If phase differences would be distributed uniformly, the coherence would equal 0, while in perfect synchronization, the coherence would equal 1.

In a third step, general adaptation behavior was explored using PL, *movement time* (MT) and *median velocity* (MV) for each half-cycle. All measures were averaged per trial and actor and compared between conditions.

## Results

### Spatial behavior

For the path length, a (2 × 3) × 2 mixed repeated measures ANOVA was performed with the within-subject factors *configuration* (congruent, incongruent) and *start delay* (zero-cycle, quarter-cycle, half-cycle), and the between-subject factor *actor* (HAMP, FREE). Prediction based comparisons by means of dependent *t*-tests (1-tailed) were performed to clarify intrapersonal differences (Field, 2009).

Path length (PL) was determined as the length of the 3D-trajectory performed during the movement interval of each cycle and direction (forwards, backwards). The median was calculated of all half-cycle trajectory lengths per trial and averaged per direction and participant.

**Figure 3:**
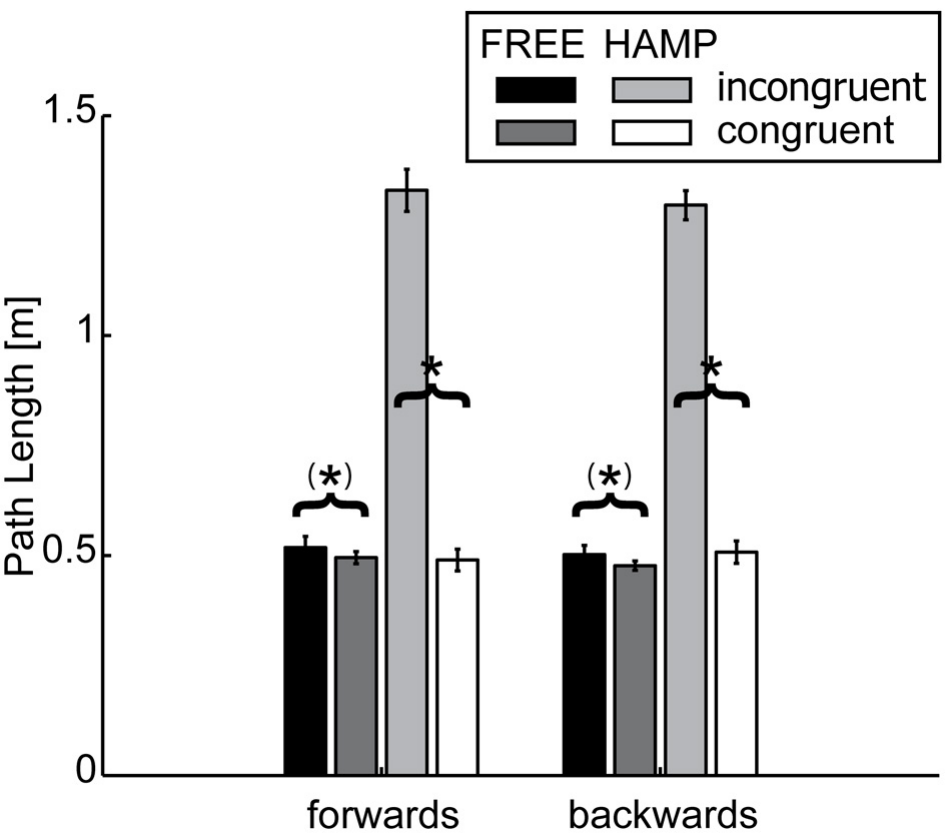
**Path Length (PL) of FREE and HAMP in both configurations**. Error bars depict the standard error of the mean over participants within one condition. In the obstacle present configuration, the hampered actor extends the trajectory to avoid possible collisions. But also the free actor slightly increases path length if HAMP has to clear the obstacle. ^*^Denotes a significance of *p* < 0.05; (^*^) stands for marginally significant results (0.05 < *p* < 0.1).

Comparison of path lengths revealed that the trajectories were significantly longer for both forwards and backwards movements in the incongruent configuration, see Figure 3 and Table 1. In general the hampered actor extended the trajectory. Although the free actor also slightly extended the path length during the incongruent configuration, this effect was only marginally significant, see Table 2.

**Table 1:** **Results of (2 × 3) × 2 mixed design repeated measures ANOVAs with the within subject factors configuration (congruent, incongruent) and start delay (zero-cycle, quarter-cycle, half-cycle) and the between subject factor person (HAMP, FREE)**.

		**Forwards/distant target**	**Backwards/close target**
PL	C	*F*_(1, 18)_ = 320.69, *p* < 0.001^*^	*F*_(1, 18)_ = 469.38, *p* < 0.001^*^
	P	*F*_(1, 18)_ = 121.31, *p* < 0.001^*^	*F*_(1, 18)_ = 209.33, *p* < 0.001^*^
	C × P	*F*_(1, 18)_ = 287.96, *p* < 0.001^*^	*F*_(1, 18)_ = 412.28, *p* < 0.001^*^
DT	C	*F*_(1, 18)_ = 2.51, *p* = 0.131	*F*_(1, 18)_ = 0.45, *p* = 0.510
	P	*F*_(1, 18)_ = 0.12, *p* = 0.730	*F*_(1, 18)_ = 0.46, *p* = 0.833
	C × P	*F*_(1, 18)_ = 9.38, *p* = 0.007^*^	*F*_(1, 18)_ = 5.40, *p* = 0.032^*^
MT	C	*F*_(1, 18)_ = 40.30, *p* < 0.001^*^	*F*_(1, 18)_ = 35.71, *p* < 0.001^*^
	P	*F*_(1, 18)_ = 3.88, *p* = 0.064	*F*_(1, 18)_ = 0.99, *p* = 0.334
	C × P	*F*_(1, 18)_ = 27.68, *p* = 0.001^*^	*F*_(1, 18)_ = 18.58, *p* = 0.001^*^
MV	C	*F*_(1, 18)_ = 85.97, *p* < 0.001^*^	*F*_(1, 18)_ = 135.01, *p* < 0.001^*^
	P	*F*_(1, 18)_ = 9.23, *p* < 0.007^*^	*F*_(1, 18)_ = 12.36, *p* < 0.002^*^
	C × P	*F*_(1, 18)_ = 85.94, *p* < 0.001^*^	*F*_(1, 18)_ = 135.18, *p* < 0.001^*^

P-values are reported to be significant

“*” *if the 2-tailed significance level p < 0.05. Statistics for start delay are not provided as they were all not significant, all p > 0.06. PL, Path Length; DT, Dwell Time; MT, Movement time; MV, Median Velocity; C, Configuration; P, Person*.

**Table 2:** **Results of pairwise directed comparisons to clarify intrapersonal behavioral differences between configurations**.

	**Actor**	**Forwards/distant target**	**Backwards/close target**
PL	HAMP	*t*_(9)_ = 18.51, *p* < 0.001^*^	*t*_(9)_ = 22.74, *p* < 0.001^*^
	FREE	*t*_(9)_ = 1.40, *p* = 0.098	*t*_(9)_ = 1.77, *p* = 0.055
DT	HAMP	*t*_(9)_ = −2.41, *p* = 0.020^*^	*t*_(9)_ = −1.58, *p* = 0.074
	FREE	*t*_(9)_ = 2.80, *p* = 0.011^*^	*t*_(9)_ = 2.56, *p* = 0.016^*^
MT	HAMP	*t*_(9)_ = 5.87, *p* < 0.001^*^	*t*_(9)_ = 5.35, *p* < 0.001^*^
	FREE	*t*_(9)_ = 3.58, *p* = 0.003^*^	*t*_(9)_ = 3.01, *p* = 0.008^*^
MV	HAMP	*t*_(9)_ = 9.87, *p* < 0.001^*^	*t*_(9)_ = 12.02, *p* < 0.001^*^
	FREE	*t*_(9)_ = 0.002, *p* = 0.499	*t*_(9)_ = −0.014, *p* = 0.495

P-values are reported to be significant

“*” if the 1-tailed significance level p < 0.05. PL, Path Length; DT, Dwell Time; MT, Movement time; MV, Median Velocity.

### Synchronization

#### Distribution of relative phase

To check whether synchronization emerged under incongruent conditions, the distribution of relative phase was calculated, see section Data Analysis and Dependant Variables. A 2 × 3 × 9 repeated measures ANOVA was performed for both configurations (congruent, incongruent) with the within subject factors *coupling* (yes, no), *start delay* (zero-cycle, quarter-cycle, half-cycle) and *phase region* (9 regions from 0 to pi).

**Figure 4:**
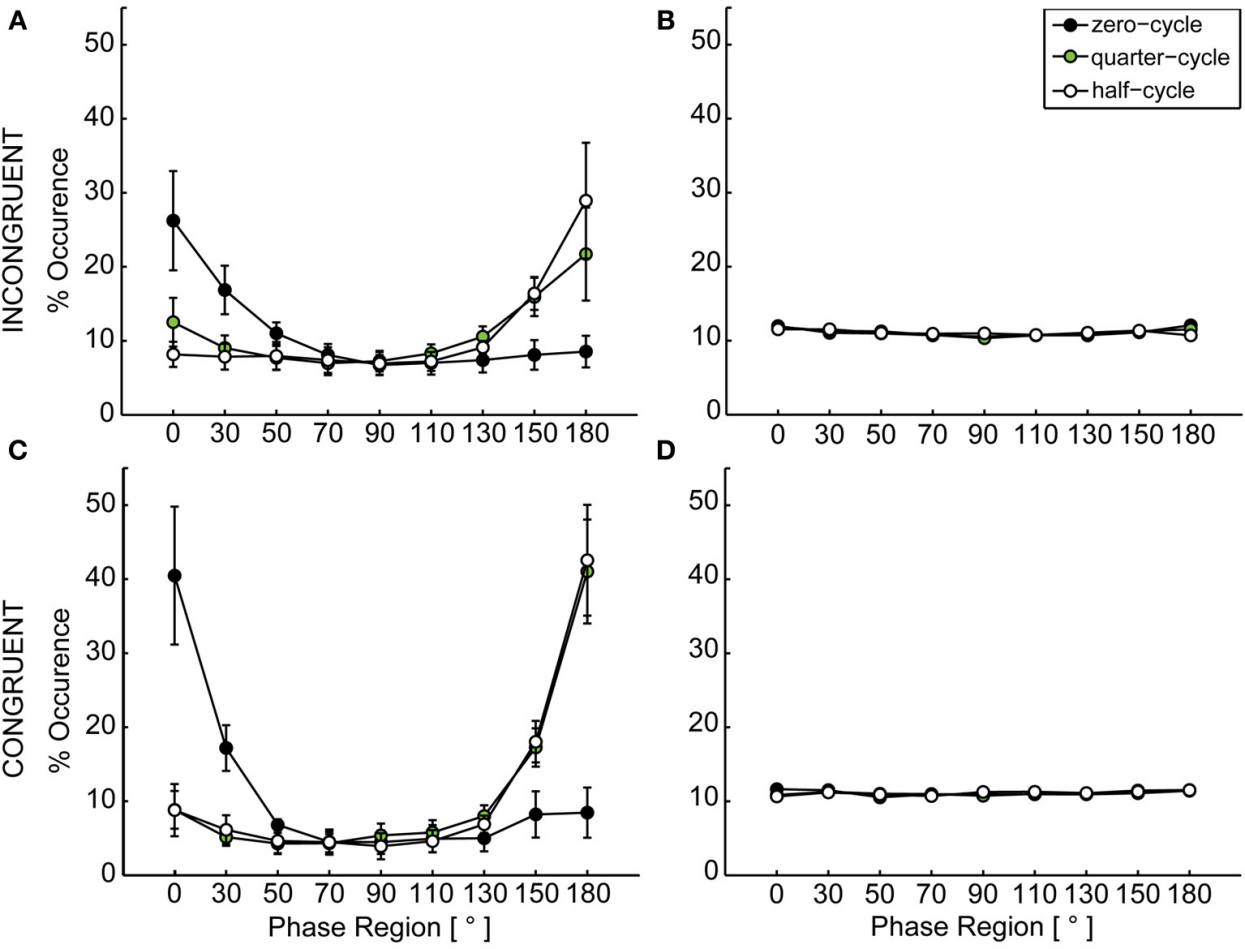
**Distribution of relative phase**. **(A)** shows the dyadically coupled data for the incongruent configuration, **(B)** the uncoupled permuted data for the incongruent case, **(C)** the dyadically coupled data for the congruent configuration and **(D)** the uncoupled permuted data for the congruent configuration. In **(A)** and **(B)**, error bars depict the standard error of the mean over participants within one condition. In **(C)** and **(D)**, the average standard error over nine phase regions was 0.61 for the incongruent and 0.52 for the conguent configuration and thus too small to be depicted. In both dyadically coupled configurations the distribution shows clear peaks at 0° (in-phase relation) and 180° (anti-phase relation). Depending on the induced start delay, participants mainly ended up in in-phase relation (after zero-cycle delay) or anti-phase relation (after quarter-cycle and half-cycle delay).

For both configurations we found a significant main effect of phase region, congruent: *F*_(8, 72)_ = 12.83, *p* < 0.001, incongruent: *F*_(8, 72)_ = 4.55, *p* < 0.001, indicating a non-uniform distribution of relative phase angle occurrence over the nine regions. Figure 4 shows the distribution of relative phase angles over phase regions resulting from the three start delay conditions. Significant interaction effects were found for coupling x phase, congruent: *F*_(8, 72)_ = 12.14, *p* < 0.001, incongruent: *F*_(8, 72)_ = 3.38, *p* = 0.002, reflecting the peaks for in-phase and anti-phase synchronization in the coupled case for both configurations. Also the phase region by start delay interaction reached significance in both configurations, congruent: *F*_(16, 144)_ = 9.02, *p* < 0.001, incongruent: *F*_(16, 144)_ = 3.41, *p* < 0.001. This is explained by the fact, that starting at the same time (zero-cycle) resulted in mainly in-phase coordination (peaks at 0°), while a shift in start delay (quarter-cycle or half-cycle) more often resulted in anti-phase coordination. The three-way interaction was also significant in both configurations, congruent: *F*_(16, 144)_ = 9.08, *p* < 0.001, incongruent: *F*_(16, 144)_ = 3.64, *p* < 0.001, which underlines that the start delay x phase region interaction was significant and present in both configurations when coupling was provided in comparison to the non-coupled case reflected by the permuted data.

#### Coherence

**Figure 5:**
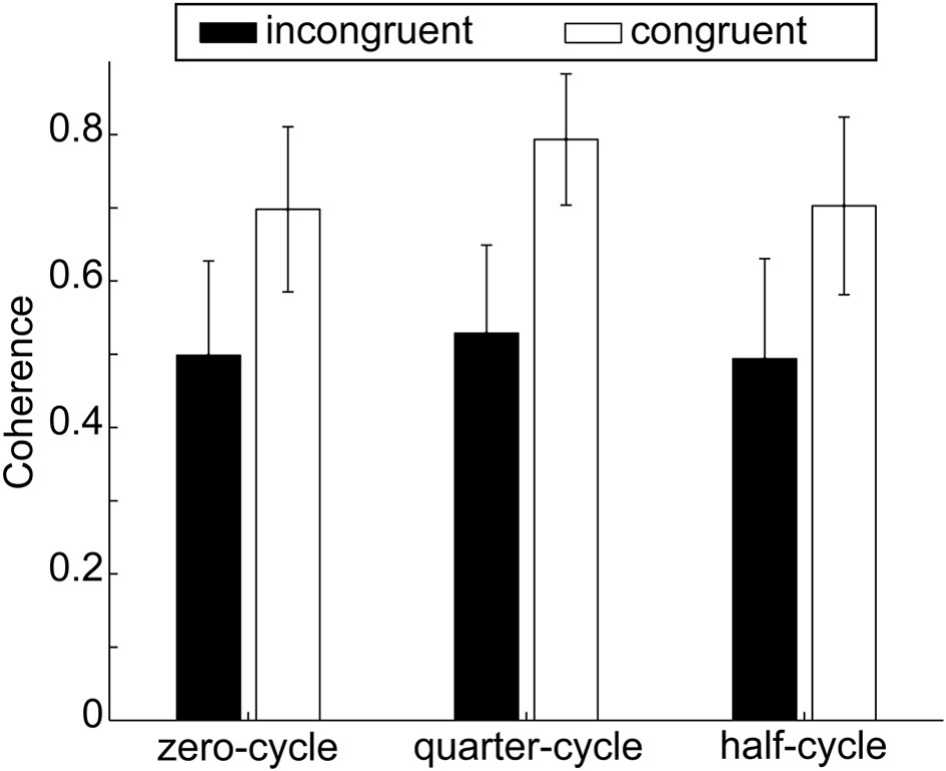
**Coherence for both obstacle configurations and in the three start delay conditions**. Error bars depict the standard error of the mean over participants within one condition. Numerically coherence is lower in the obstacle present configuration; however the difference between conditions was not significant.

For finding the degree to which people were correlated, the cross spectral coherence was calculated for each configuration, see section Data Analysis and Dependent Variables. A 2 × 3 repeated measures ANOVA on the within subject factors configuration (congruent, incongruent) and start delay (zero-cycle, quarter-cycle, half-cycle) did not yield any significant effect, all *p* > 0.1. Nevertheless the coherence was numerically higher when the obstacle was absent in all start delay conditions, see Figure 5. In the congruent configuration, coherence was 0.73 ± 0.13 (*SE*) while in the incongruent configuration coherence was only found to be 0.51 ± 0.11 (*SE*).

### Temporal behavior

In section Spatial Behavior it was reported that the obstacle caused prolonged trajectories for the hampered actor, while the free actor only marginally extended the trajectories during incongruent trials. Nevertheless synchronization emerged in both configurations. Thus, a certain temporal adjustment must have taken place. Else, if no temporal adjustment happened, synchrony would not emerge because the time that is required to perform the prolonged trajectory naturally extends the time to perform the shorter trajectory at the same velocity. With this, a continuous drift in phase relation would be caused and the distribution of relative phase would be equal to the permuted data, see for example Figure 4B.

In general, there are several possibilities for establishing synchronization in an incongruent configuration. In this context different aspects can be regarded: (i) in which temporal period the adjustment happens and (ii) who makes the adjustments. Regarding temporal period, there are three possibilities for adaptation. First, people could adapt during their movement period between targets, second they could wait for each other in either one or both target points, or third, they could apply both strategies mentioned before and continuously adapt.

Regarding the aspect of who is adjusting, again three possibilities apply: one or the other actor could take over the whole load and adjust the movement to the other actor. More specifically this means, that if the free actor does not change behavior, the hampered actor would have to take all the effort. Thus, he/she could increase movement velocity and/or reduce dwell times in the targets to keep up with the free actor whose trajectory is only marginally extended. If, as the second possibility, the free actor takes over all the adjustment effort, then one would expect a reduction in his/her movement velocity or an increase in dwell times—as an extension of the trajectory is already shown to be only marginal there. However, if as a third possibility, mutual adaptation and thus a joint effort is required to establish synchronization (see Konvalinka et al., 2010), we should find adaptation in the movement profiles of both actors—even when only one actor has to clear the obstacle.

For all temporal measures (2 × 3) × 2 mixed repeated measures ANOVAs were performed with the within-subject factors *configuration* (congruent, incongruent) and *start delay* (zero-cycle, quarter-cycle, half-cycle), and the between-subject factor *actor* (HAMP, FREE). Prediction based comparisons by means of dependent *t*-tests (1-tailed) were performed to clarify intrapersonal differences (Field, 2009).

#### Dwell time (DT) and movement time (MT)

Dwell time (DT) was determined as the time participants spent in one of the targets. This time was determined by the entry and exit indices described in section Data Preparation. The remaining time in between was considered as the time in which participants were actually moving their arm forwards or backwards. These time periods are called movement time (MT) in the following.

**Figure 6:**
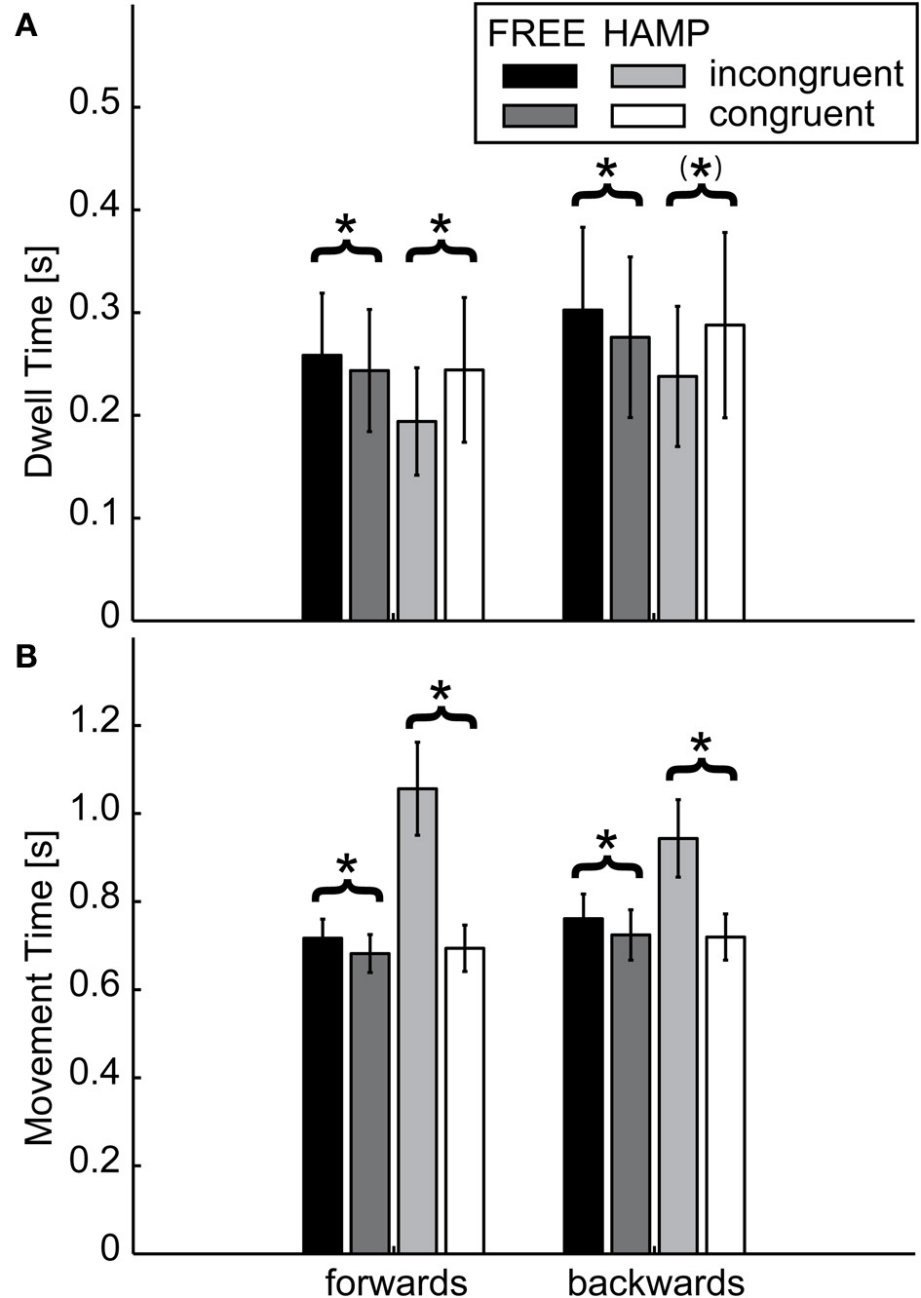
**(A)** Dwell Times and **(B)** Movement Time of FREE and HAMP in both configurations. Error bars depict the standard error of the mean over participants within one condition. While the hampered actor increases movement time to account for the prolonged trajectory, the free actor also moves slower if the obstacle is present. However regarding the time spent in target positions, the hampered actor dwells less if an obstacle is present, while the free actor increases dwell times. Thus, the free actor “waits” for the interaction partner and acts more predictable. ^*^Denotes a sgnificance of *p* < 0.05; (^*^) stands for marginally significant results (0.05 < *p* < 0.1).

Regarding DT, the only significant effect was a configuration x actor interaction in both targets, see Table 1. During incongruent configuration, the actor who had to clear the obstacle significantly reduced his/her dwell time, while the actor without the obstacle significantly increased the dwell time compared to the congruent case, see Figure 6. This means that during the dwelling phase, a joint effort is undertaken.

For MT, a significant main effect of configuration was obtained for both movement directions, see Table 1. Also the main effect of actor reached significance in both movement directions. However both main effects can be explained by the highly significant configuration x actor interaction indicating a much higher movement time for HAMP during incongruent configuration. Furthermore, pairwise comparison showed that FREE also significantly increased movement time during forwards and backwards movement if the obstacle was present, see Table 2.

Overall, no main or interaction effect was obtained for start delays, all >0.06, indicating that the behavioral differences found here apply for both the emergence of in-phase and anti-phase synchronization.

#### Median velocity

Velocity was calculated from the distance between data points in Cartesian coordinates. For smoothing the data, a fourth order low-pass Butterworth filter with a cut-off frequency of 10 Hz was applied. The resulting phase shift was corrected by applying the same filter reversely. For each forward and backward movement the median velocity (MV) was determined and averaged over each trial and dyad per condition.

**Figure 7:**
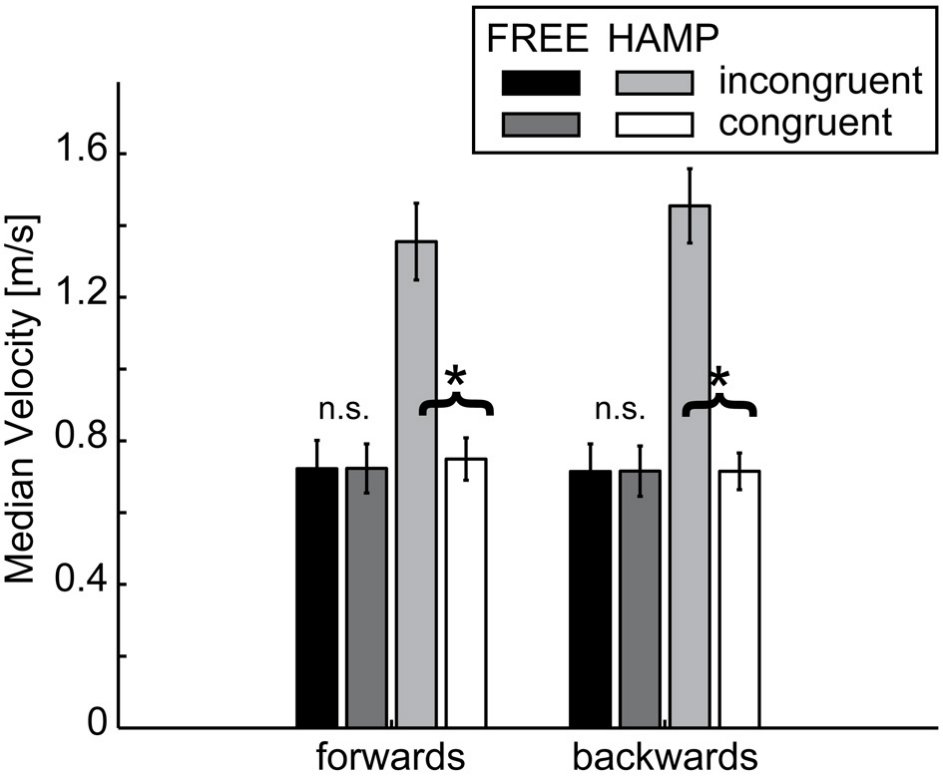
**Median Velocity of FREE and HAMP in both configurations**. Error bars depict the standard error of the mean over participants within one condition. The hampered actor on average increases movement velocity while passing the obstacle while the free actor's velocity is not affected if an obstacle is present in the overall configuration. ^*^Denotes a significance of *p* < 0.05; n.s. is not significant.

A significant main effect was observed for configuration in both directions indicating a higher MV during the incongruent condition, see Figure 7 and Table 1. However, also the factor actor yielded a significant main effect in both movement directions, indicating a higher MV for the actor who had to clear the obstacle, see Table 1. Both effects can be explained by the significant configuration x actor interaction: in both forward and backward movements, HAMP moved significantly faster if the obstacle was present, while the MV of FREE was not affected.

No main or interaction effect was found for start delays, all *p* > 0.15. This indicates that MV has no influence on the emergence of either in-phase or anti-phase synchronization.

## Discussion

With the present study we investigated the question whether movement synchronization also emerges between target-directed arm movements of two people when their performed trajectories are incongruent. An obstacle was included into one actor's workspace which caused a prolongation of trajectory. Due to the incongruence of movement trajectories of both actors, we expected movement interference to emerge which could cause synchronization to break down. On the other side, also the effort of obstacle avoidance might lead to non-synchronous behavior.

### Movement synchronization

If movement trajectories are incongruent during dyadic interaction in a target-directed task, movement synchronization still emerges as indicated by peaks in the distribution of relative phase around 0 and 180° compared to the non-synchronous case. Numerically, the cross spectral coherence was smaller in the incongruent configuration in which the obstacle was present. As the difference in coherence is not significant, this finding might mainly reflect the higher effort which comes with the need to enable a useful strategy for compensating with the more difficult situation.

### Movement interference

In the incongruent case, when the hampered actor had to clear the obstacle and therefore extended the trajectory, the trajectory of the free actor was also marginally extended.

Movement interference of motor contagion theories predict that if observing somebody else performing an action incongruent to our own action, our own action performance is biased and variability is increased (Kilner et al., 2003; Stanley et al., 2007). Following this idea, one could argue that the small increase of path length of the free actor in obstacle-present trials only happened due to the aforementioned effect and has nothing to do with movement adjustment. However, if the prolongation of the trajectory only appeared due to movement interference, the dwelling behavior as an adaptation process would lose its purpose. An alternative explanation comes from the idea of the so-called *rhythmic movement interference* (RMI), which even predicts increased deviations while observing incongruent movements (incongruent trajectories). These deviations however are not thought to be a problem to the emergence of synchronization (as the motor contagion theory would predict) but rather the enabler for it. The RMI states that while observing somebody performing movements incongruent to the own ones, additional degrees of freedom are liberated by deviating from one's original plane. These additional degrees of freedom stabilize coordination in situations in which it would otherwise be unstable (Fink et al., 2000; Milliex et al., 2005; Richardson et al., 2009; Romero et al., 2012). In this context, Fink et al. (2000) showed that when limiting a bimanual pendulum swinging task to a single plane, transitions from anti-phase to in-phase synchronization (the more stable state) appeared. These transitions were absent if the swinging plane was not restricted. Instead, deviations from the instructed plane were observed, which were obviously used to sustain anti-phase coordination. For the present study this implies that the free actor could have adjusted to the higher variability of the hampered actor in the obstacle-present case by showing higher deviations from the instructed direction of motion (forwards/backwards) and vice versa. Thus, the RMI would allow for explaining how movement synchronization was enabled and coherence was only slightly decreased.

Another interesting idea for explaining the adaptation of the free actor to the hampered actor in space and time is the notion of hand path priming by the use of spatio-temporal forms (van der Wel et al., 2007). If an obstacle is present in the shared peri-personal space, the free actor might take on the predefined spatio-temporal forms that are defined for obstacle avoidance and with this prepare for accounting to the hampered actor's behavior. This idea is supported by findings of Castiello (2003). In an experiment in which one person had to reach and grasp an object in the presence of a distractor, the subsequent similar action without a distractor of the person previously observing this action was biased in the same way as the movement of the first person with distractor. Castiello also showed, that both persons paid similar attention to the distractor, as indicated by eye movements, irrespective of whether they were affected by it. Thus, in the present study the obstacle might afford a different spatio-temporal form—also for the free actor and regardless of the need to clear it, only because it is there.

The fact that the spatial adaptation of the free actor was not very striking might be owed to the relative distance between participants. The shared workspace was relatively close to the border of each actor's peri-personal space (Previc, 1998) and thus the movements of the free actor were not as affected as they might have been in closer interaction. Furthermore, participants were sitting opposite to each other, not next to each other. This implies that the deviations induced by the obstacle in the movement trajectory of the hampered actor might have been hard to guess because they had to be estimated frontally, not from the side.

### Mutual adaptation and joint action

Nevertheless, despite all observed difficulties, synchronization emerged when an obstacle was present through adaptation by one or both interaction partners. The hampered actor had to extend the movement trajectory, and thus movement trajectories of the dyad were incongruent. Here, compensation for the prolonged trajectory, led to an increase of movement velocity. This is especially interesting in the light of known obstacle avoidance strategies which show that if an obstacle has to be cleared, movement velocity is decreased in order to increase accuracy of the movement and avoid potential collisions with the obstacle (Tipper et al., 1997; Coppard et al., 2001; Chapman and Goodale, 2008). However these findings derive from discrete and non-repetitive tasks in which participants were not in an interaction situation at the same time.

Vesper et al. (2009) showed that while jointly building a marble track by moving wooden building bricks from a defined start to a defined target position, movement velocity was increased compared to performing the same task alone. Similar to the present study, a decreased movement time and an increased path length (transport path) was observed during the joint action condition compared to single action. The authors argued that the increase of speed and the increased deviation during joint action in comparison to single action might be explained by the intention of participants to clear the joint workspace as soon as possible in order to clear the space for the interaction partner. Following this, also interaction in close space may be treated as dealing with obstacles (the other actor) and thus the reaction could be a constant obstacle/collision avoidance behavior during joint tasks. However, Vesper et al. only focused on the behavior of one person in a joint task and the dynamics of the interaction were not regarded. In the present study the interaction dynamics were one focus and it was shown, that synchronization patterns emerge between interaction partners even if one interaction partner was dealing with obstacle avoidance. Here, the free actor and his/her movements were in the direct field of view of the hampered actor when reaching over the obstacle. Therefore it can also be argued that because both actors were engaged in a repetitive task with the possibility of observing each other, they could not avoid synchronizing their movements (Issartel et al., 2007; Schmidt et al., 2007). In this case, the obstacle avoidance task would be the secondary task, and the discrete obstacle avoidance behavior (slowing down) was sacrificed and higher effort was applied to fulfill the needs (speed up) of successful synchronization. Support for this notion comes from Doumas et al. (2008), who explored movement synchronization in a bimanual repetitive finger tapping task. In their study, taps had to be synchronized to a metronome and had to be performed at different movement amplitudes. If at the same time interval tapping amplitude was higher, movement velocity was increased in order to keep track with timing constraints from the metronome. Thus, when the amplitude has to be increased, a natural reaction is to speed up in order to remain in synch.

In a similar way, movement synchronization with another actor bears temporal constraints. If an actor wants to keep track with an interaction partner who can perform his/her movements at lower amplitude, the actor has to speed up to keep track with the timing demands of synchronous movements. Therefore, the obstacle in the present study would simply be treated as a “higher amplitude generator” which has to be compensated to reach the joint unintentional goal, namely movement synchronization.

What adds to this argumentation is the finding that also the free actor—who was in no need to react to the obstacle—unintentionally, took part in the compensation process. Participants on the FREE side slightly increased their path length and with this reached a higher movement time if the hampered actor had to clear an obstacle, by on average keeping movement velocity constant. With this strategy, the free actor was always providing a predictable behavior and potentially enabled a successful adaptation of the interaction partner.

On top of that, the free actor increased dwell times in both targets and with this “waited” for the interaction partner during the incongruent configuration, while the hampered actor decreased dwell times in both targets when an obstacle was present. The adjustment of dwell times observed in both actors might also be related to an adjustment of the perceptual center of the perceived event (p-center hypothesis, Morton et al., 1976; Aschersleben, 2002). The p-center hypothesis assumes that each event that is extended over time has a perceptual center that differs from the onset of the stimulus. It is also stated, that its position in time depends on stimulus duration (among others). This also means that if the stimulus duration is increased, then there is a bigger delay between stimulus onset and its perceptual center. This can also be used for synchronization: if the free actor increases dwell times, the hampered actor has more time to estimate the perceptual center and can adjust his/her movements accordingly. However this would only apply if the event which is used to synchronize with each other is the perceptual center of a time period—in contrast to its onset.

Taken together, it seems as if the hampered actor mainly compensates for the increased movement trajectory, while the free actor tries to make this adjustment process as easy as possible. With this, the unintentional goal to synchronize in an incongruent scenario can be reached. In literature it was also claimed, that people actively and mutually adapt to each other's behavior in order to synchronize their movements (i.e., Konvalinka et al., 2010). Adding to this however, our results show that people do not only mutually represent the task (Frith and Frith, 2010; Obhi and Sebanz, 2011; Wenke et al., 2011), they also assign different roles to each other depending on the needs of the task and in order to compensate for the increased effort induced by the obstacle. While one actor operates as compensator, the other one accommodates these compensatory movements by making himself as predictable as possible. This means, that movement synchronization in an incongruent case is not only a merely emerging behavior, it also bears features of a joint action task, in which complementary actions have to be fulfilled in order to reach accomplishment.

## Conclusion

If the movement trajectories of people engaged in a repetitive target-directed tapping task are incongruent, movement synchronization still emerges. Moreover, if the trajectory of one actor is disturbed by an obstacle, the regular obstacle avoidance strategies (decreased velocity) do not apply—presumably due to prioritization of movement synchronization with the partner. Therefore, different adaptation roles are assigned between interaction partners: while one actor has to deal with a more difficult task (obstacle avoidance), the interaction partner aims to be as predictable as possible by adapting dwell times and maintaining speed. In summary, if a simple component like an obstacle is added to a target-directed tapping task in a shared workspace, movement synchronization not merely emerges between interaction partners; it also requires complementary actions like any higher level joint action task.

## Author contributions

Tamara Lorenz, Björn N. S. Vlaskamp, and Sandra Hirche defined the research question on synchronization in goal-directed tasks. Tamara Lorenz, Björn N. S. Vlaskamp, and Anna-Maria Kasparbauer designed the experiment. Anna-Maria Kasparbauer, Tamara Lorenz, and Alexander Mörtl performed the experiment. Tamara Lorenz, Anna-Maria Kasparbauer, Björn N. S. Vlaskamp, and Alexander Mörtl analyzed the data. Tamara Lorenz, Anna-Maria Kasparbauer, Björn N. S. Vlaskamp, Alexander Mörtl, and Sandra Hirche wrote the paper. Björn N. S. Vlaskamp and Sandra Hirche supervised the project.

### Conflict of interest statement

The authors declare that the research was conducted in the absence of any commercial or financial relationships that could be construed as a potential conflict of interest.
